# Prevalence of cannabis use in people with psychosis in KwaZulu-Natal, South Africa

**DOI:** 10.4102/sajpsychiatry.v28i0.1927

**Published:** 2022-10-21

**Authors:** Khanya Mona, Vuyokazi Ntlantsana, Andrew M. Tomita, Saeeda Paruk

**Affiliations:** 1 Discipline of Psychiatry, School of Clinical Medicine, College of Health Sciences, University of KwaZulu-Natal, Durban, South Africa; 2 Department of Psychiatry, KwaZulu Natal Research Innovation and Sequencing Platform (KRISP), College of Health Sciences, University of KwaZulu- Natal, Durban, South Africa; 3 Discipline of Psychiatry, Centre of Rural Health, School of Nursing and Public Health, College of Health Sciences, University of KwaZulu Natal, Durban, South Africa

**Keywords:** schizophrenia spectrum disorder, psychotic disorders, cannabis use, South Africa, alcohol use

## Abstract

**Background:**

There is a high prevalence of cannabis use in patients with schizophrenia spectrum and other psychotic disorders, with comorbid cannabis use in this population being associated with poorer long-term outcomes.

**Aim:**

To determine the prevalence of cannabis use in patients with a schizophrenia spectrum and other psychotic disorders.

**Setting:**

The study was conducted at a psychiatric hospital in Durban, KwaZulu-Natal Province, South Africa.

**Methods:**

A review of clinical records of patients admitted to the hospital for the period, June 2018 to June 2020, was conducted.

**Results:**

A total of 370 clinical records were reviewed, of which 48.9% reported current and 51.1% lifetime cannabis use. Being male was significantly associated with current and lifetime cannabis use (OR = 4.90, 95% CI 2.49–9.62 and OR = 6.27, 95% CI 3.28–11.95, respectively). Current alcohol use was also associated with current cannabis use (CCU) (OR = 3.06, 95% CI 1.78–5.28), and age 45 years and older was associated with a lower odds of cannabis use (OR = 0.30, 95% CI 0.09–0.96). Forty-eight per cent of participants were admitted three or more times, and readmission was associated with cannabis use (*p* = 0.01). There was a lack of association between cannabis use, readmission and human immunodeficiency virus (HIV) status, after controlling for variables such as alcohol use and gender.

**Conclusion:**

Almost 50% of people admitted with schizophrenia spectrum and other psychotic disorders have comorbid current and lifetime cannabis use. There is a need for dual diagnosis units to address comorbid substance use in people with psychotic disorders, as it leads to poorer outcomes.

**Contribution:**

The study found that there is a high prevalence of cannabis use in people with psychosis. Therefore, it is imperative that we revise treatment programs in our psychiatric units and there is an urgent need for dual diagnosis programs that address substance use in this group of patients.

## Introduction

Illicit substance use is a worldwide problem and contributes significantly to the global burden of disease.^1^ The United Nations office on Drugs and Crime world drug report (2020) found that cannabis is one of the most commonly used drugs, with approximately 192 million users, of whom 35 million meet the criteria for cannabis use disorder.^2^

Schizophrenia is a chronic and severe mental illness that is characterised by positive, negative, affective and cognitive symptoms. It runs an often-disabling course and places a considerable burden on patients, their families, the healthcare system and society at large.^3,4^ Comorbid cannabis use in schizophrenia spectrum and other psychotic disorders is associated with a more severe course of illness. Cannabis users with psychosis have an earlier onset of illness, increased symptom severity, poorer adherence to psychiatric medication, poorer functional outcomes, higher relapse rates and longer hospital stays compared with non-users.^5,6,7,8,9,10,11^ In addition, people with schizophrenia spectrum and other psychotic disorders and comorbid cannabis use tend to have poorer psychosocial functioning, homelessness, violence, incarceration and increased burden of care for families.^4,12,13,14^

The literature suggests a high prevalence of comorbid cannabis use among people with psychotic disorders,^15,16^ with the concurrent use of other substances, for example alcohol, also having been reported.^10^ In a meta-analysis of 35 studies of adults presenting with first episode psychosis (FEP), 33.7% reported cannabis use, 30.7% of outpatients with FEP reported past month cannabis use and 34.7% met the criteria for lifetime cannabis abuse or dependence.^17^ Studies conducted in the United States of America (USA) and Canada found that cannabis use is twice as common in patients with severe mental illness, such as schizophrenia, compared to the general population.^18^ The prevalence of cannabis use in South African populations with psychotic illness has seen reported to be between 37% and 68%, depending on the setting and population.^19,20,21^ A Cape Town study found the most common substance use disorder associated with psychosis was cannabis use disorder, with a prevalence of 34.3%.^15^

Recent studies show that the use of high potency cannabis increases the risk of FEP and subsequent schizophrenia, and that psychosis and prodromal schizophrenia symptoms may also exacerbate cannabis use.^22^ Genetics and environmental factors play a role in this bidirectional relationship between psychosis and cannabis use.^23^ Various socio-demographic and clinical factors may be associated with cannabis use among people living with psychosis. International studies found that cannabis use in patients with psychosis was predominantly in males, younger in age, of a low education level and socio-economic status.^24,25^ Data from South Africa (SA) and other African countries have shown similar findings, with unemployment, being single, an inpatient status, and recurrent admissions being additional factors associated with cannabis use.^5,15,16,19,26,27^

Hence we note a high comorbidity between cannabis use and psychosis, and that the dual diagnosis of a cannabis use and psychotic disorder is a poor prognostic factor. A study conducted in India had similar findings where cannabis use had a high prevalence for psychotic disorder and this co-occurrence is a poor prognostic factor.^28^ In KwaZulu-Natal (KZN) Province, SA, Paruk et al.^20^ found a cannabis use prevalence rate of 68.8% in the adolescent psychotic population, while Burns and colleagues found a prevalence rate of 35% in the adult population of the same province.^29^ These studies were all conducted about a decade ago, before the personal use of cannabis was decriminalised in SA.^30^ The factors associated with comorbidity in the African setting may also differ from elsewhere, as this may be influenced by cultural and environmental differences between countries and availability of cannabis, while the decriminalisation and legalisation policies on cannabis may have an impact on the patterns of use.

This research aimed to describe the socio-demographic and clinical profiles of patients admitted with schizophrenia spectrum and other psychotic disorders to a psychiatric hospital. The study also aimed to determine the prevalence and pattern of cannabis use (current vs. lifetime) in this population and to describe any associations between cannabis use and socio-demographic and clinical variables.

## Aim

The association between cannabis use and psychosis has been established in recent years, but there has been a move to decriminalise personal cannabis use globally and in SA; hence the authors aimed to review the prevalence of current and lifetime cannabis use in people living with psychosis.

## Methods

A retrospective chart review was conducted at a tertiary psychiatric hospital that offers psychiatric services in the *eThekwini* District, KZN Province, SA.

### Study population

All in-patients 13 years and older meeting the *DSM-5* criteria for schizophrenia spectrum and other psychotic disorders who were admitted between 01 June 2018 and 31 June 2020 were included. Clinical records were obtained from the hospital registry of admissions, with those with incomplete or missing data being excluded from the study.

### Data collection

A structured socio-demographic and clinical data questionnaire was used to collate data from the clinical records, the former including age, gender, marital status, education level, current employment status and place of residence. The clinical data collected included family history of psychotic and non-psychotic illness and substance use, Diagnostic and Statistical Manual of Mental Disorders (DSM-5) diagnostic categories,^31^ age at first admission and duration of illness. Substance use history, current and lifetime use, duration of hospitalisation, number of hospitalisations and human immunodeficiency virus (HIV) status data were also collated. Cannabis use history was based on patient and family report, and the history of other illicit substances was also extracted. All data extracted from the clinical files was collected by the principal investigator, captured manually into a data collection sheet and later captured electronically onto Research Electronic Data Capture (REDCAP) database.^32^

For the study, the following terminology is defined based on a review of the literature and consensus by the research team:

- Schizophrenia spectrum and other psychotic disorders include schizophrenia, other psychotic disorders and schizotypal personality disorder and are defined by the presence of at least one of the following domains: hallucinations, delusions, disorganised thinking or behaviour and negative symptoms.^31^
- Lifetime cannabis use: A positive report of lifetime cannabis exposure before or after seeking treatment.
- Current cannabis use (CCU): Cannabis use in the 3 months prior to first presentation for treatment.
- No cannabis use (NCU): Not having any lifetime cannabis exposure.^33^
- Substance use: A positive report of lifetime substance exposure before or after seeking treatment.
- Substance use disorder: A maladaptive pattern of substance use that results in behavioural, psychical and psychological symptoms and causes distress or impairment.^31^

### Data analysis

Stata version 15.1 was used to analyse the data, with descriptive statistics, such as frequencies and percentages, being used to summarise the results. McNemar’s chi-square test was used to test for associations between current and lifetime cannabis use and patient socio-demographic and clinical characteristics. Logistic regression models were used to test for associations cannabis use, controlling for socio-demographic and clinical variables such as age, gender, number of readmissions and HIV status, with the significance level being set at *p* = 0.05.

### Sample size

Davis et al.^34^ in their study of patients with serious psychiatric illness such as schizophrenia in KZN, found the prevalence of cannabis use to be at 49.4%. Using the formula *n* = z^2^p(1−p)/d^2^ (*n*: sample size, z: standard deviation set at 95% standard value of 1.96, p: prevalence and d: error margin), the researchers aimed to reach a target sample size of 349 participants, which was adequate to achieve a 95% confidence interval at an error margin of 5.25% in this study.^35^

### Ethical considerations

Ethical approval was obtained from the Biomedical Research Ethics Committee of the University of Kwa-Zulu Natal (BREC/00002640/2021) and the Kwa-Zulu Natal Department of Health. The study is a retrospective chart review and therefore did not require consent from participants.

## Results

### Patient socio-demographic profile

A total of 370 patient clinical records were included in the study, with two being excluded because of inadequate information. The socio-demographic and clinical information is presented in Table 1; 66.2% of participants were between the ages 16 and 34 years; most were male (70.5%), unmarried (93.9%), had a highest level of education of grade school (88.0%) and were unemployed (89.2%). The median duration of the psychotic illness was three years; 48% had been admitted three or more times, while the current and lifetime cannabis use was at 48.9% and 51.1%, respectively.

**TABLE 1:** Socio-demographic and clinical variables of people admitted with schizophrenia spectrum and other psychotic disorders.

Variable	Levels	Overall			
		*n*	%	Median	IQR
Age categories	16–24	117	31.6	-	-
	25–34	128	34.6	-	-
	35–44	59	15.9	-	-
	45+	66	17.8	-	-
Gender	Female	109	29.5	-	-
	Male	261	70.5	-	-
Marital status	Single or divorced or widowed	341	93.9	-	-
	Married	22	6.1	-	-
Educational level	No formal education or remedial school	10	2.8	-	-
	Mainstream school up to grade 12	315	88.0	-	-
	Tertiary Level	33	9.2	-	-
Current employment status	Unemployed	321	89.2	-	-
	Employed (formal or informal sector)	39	10.8	-	-
Family history for psychosis	No	304	82.2	-	-
	Yes	66	17.8	-	-
Family history for non-psychotic mental illness	No	345	93.2	-	-
	Yes	25	6.8	-	-
Family history for substance use disorder	No	353	95.4	-	-
	Yes	17	4.6	-	-
Duration of illness (in months)	-	-	-	36	11–156
Number of hospitalizations and admissions in the past	None	94	25.6	-	-
	1–2 readmissions	97	26.4	-	-
	3 or more readmissions	176	48.0	-	-
HIV status	Positive	44	12.1	-	-
	Negative	209	57.6	-	-
	Unknown	110	30.3	-	-
Current alcohol Use	No	231	62.8	-	-
	Yes	137	37.2	-	-
Current cannabis use	No	188	51.1	-	-
	Yes	180	48.9	-	-
Current cocaine and cocaine containing substance use	No	356	96.7	-	-
	Yes	12	3.3	-	-
Current heroine and heroine containing substance use	No	353	95.9	-	-
	Yes	15	4.1	-	-
Lifetime alcohol use	No	202	54.9	-	-
	Yes	166	45.1	-	-
Lifetime cannabis use	No	155	42.1	-	-
	Yes	213	57.9	-	-
Lifetime cocaine and cocaine containing substance use	No	355	96.5	-	-
	Yes	13	3.5	-	-
Lifetime heroine and heroine containing substance use	No	351	95.4	-	-
	Yes	17	4.6	-	-

HIV, human immunodeficiency virus.

### Factors associated with cannabis use

Table 2 details the socio-demographic and clinical variable based on current and lifetime cannabis use. Being in the 14–24 and 25–34-year age groups, male gender was significantly associated with current and lifetime cannabis use (*p* = < 0.001). Not having a partner, grade school as the highest level of education in mainstream school, shorter duration of illness, negative HIV status was also associated with current and lifetime cannabis use. A history of three or more admissions was associated with lower prevalence CCU compared to no previous admission.

**TABLE 2:** Association between socio-demographic and clinical variables of people admitted with schizophrenia spectrum and other psychotic disorders and cannabis use.

Variable	Levels	No current cannabis (*n* = 188)				Current cannabis (*n* = 180)				*p*-value for *X*^2^	No lifetime cannabis (*n* = 155)				Lifetime cannabis (*n* = 213)				*p*-value for *X*^2^
		*n*	%	Median	IQR	*n*	%	Median	IQR		*n*	%	Median	IQR	*n*	%	Median	IQR	
Age categories	16–24	43	22.9	-	-	73	40.6	-	-	< 0.001	33	21.3	-	-	83	39.0	-	-	< 0.001
	25–34	51	27.1	-	-	76	42.2	-	-	-	39	25.2	-	-	88	41.3	-	-	-
	35–44	43	22.9	-	-	16	8.9	-	-	-	35	22.6	-	-	24	11.3	-	-	-
	45+	51	27.1	-	-	15	8.3	-	-	-	48	31.0	-	-	18	8.5	-	-	-
Gender	Female	88	46.8	-	-	20	11.1	-	-	< 0.001	85	54.8	-	-	23	10.8	-	-	< 0.001
	Male	100	53.2	-	-	160	88.9	-	-	-	70	45.2	-	-	190	89.2	-	-	-
Marital status	Single or divorced or widowed	170	91.4	-	-	169	96.6	-	-	0.04	139	90.3	-	-	200	96.6	-	-	0.01
	Married	16	8.6	-	-	6	3.4	-	-	-	15	9.7	-	-	7	3.4	-	-	-
Educational level	None or remedial school (high level or grade)	8	4.5	-	-	2	1.1	-	-	0.02	8	5.5	-	-	2	0.9	-	-	0.01
	Mainstream- highest grade	149	83.2	-	-	165	92.7	-	-	-	119	81.5	-	-	195	92.4	-	-	-
	Tertiary level	22	12.3	-	-	11	6.2	-	-	-	19	13.0	-	-	14	6.6	-	-	-
Current employment status	Unemployed	163	89.6	-	-	157	88.7	-	-	0.79	131	87.9	-	-	189	90.0	-	-	0.53
	Employed (formal or informal sector)	19	10.4	-	-	20	11.3	-	-	-	18	12.1	-	-	21	10.0	-	-	-
Family historyfor psychosis	No	151	80.3	-	-	151	83.9	-	-	0.37	125	80.6	-	-	177	83.1	-	-	0.54
	Yes	37	19.7	-	-	29	16.1	-	-	-	30	19.4	-	-	36	16.9	-	-	-
Family history for non-psychotic mental illness	No	173	92.0	-	-	170	94.4	-	-	0.36	142	91.6	-	-	201	94.4	-	-	0.30
	Yes	15	8.0	-	-	10	5.6	-	-	-	13	8.4	-	-	12	5.6	-	-	-
Family history for substance use disorder	No	177	94.1	-	-	174	96.7	-	-	0.25	144	92.9	-	-	207	97.2	-	-	0.05
	Yes	11	5.9	-	-	6	3.3	-	-	-	11	7.1	-	-	6	2.8	-	-	-
Duration of illness (in months)		-	-	60	12–192	-	-	24	11–96	< 0.001	-	-	60	11–192	-	-	24	11–108	0.002
Number of hospitalizations and admissions in the past	None	40	21.4	-	-	53	29.6	-	-	0.01	38	24.7	-	-	55	25.9	-	-	0.06
	1–2 readmissions	42	22.5	-	-	55	30.7	-	-	-	32	20.8	-	-	65	30.7	-	-	-
	3 readmissions	105	56.1	-	-	71	39.7	-	-	-	84	54.5	-	-	92	43.4	-	-	-
HIV status	Positive	32	17.4	-	-	12	6.7	-	-	0.01	30	19.7	-	-	14	6.7	-	-	<0.001
	Negative	96	52.2	-	-	112	62.9	-	-	-	74	48.7	-	-	134	63.8	-	-	-
	Unknown	56	30.4	-	-	54	30.3	-	-	-	48	31.6	-	-	62	29.5	-	-	-
Current alcohol use	No	146	77.7	-	-	85	47.2	-	-	< 0.001	122	78.7	-	-	109	51.2	-	-	<0.001
	Yes	42	22.3	-	-	95	52.8	-	-	-	33	21.3	-	-	104	48.8	-	-	-
Current cocaine and cocaine containing substance use	No	186	98.9	-	-	170	94.4	-	-	0.02	154	99.4	-	-	202	94.8	-	-	0.02
	Yes	2	1.1	-	-	10	5.6	-	-	-	1	0.6	-	-	11	5.2	-	-	-
Current heroine and heroine containing substance use	No	127	67.6	-	-	70	39.1	-	-	< 0.001	155	100.0	-	-	198	93.0	-	-	< 0.001
	Yes	61	32.4	-	-	109	60.9	-	-	-	0	0.0	-	-	15	7.0	-	-	-
Lifetime alcohol use	No	125	66.5	-	-	77	42.8	-	-	< 0.001	112	72.3	-	-	90	42.3	-	-	< 0.001
	Yes	63	33.5	-	-	103	57.2	-	-	-	43	27.7	-	-	123	57.7	-	-	-
Lifetime cocaine and cocaine containing substance use	No	185	98.4	-	-	170	94.4	-	-	0.04	153	98.7	-	-	202	94.8	-	-	0.047
	Yes	3	1.6	-	-	10	5.6	-	-	-	2	1.3	-	-	11	5.2	-	-	-
Lifetime heroine and heroine containing substance use	No	186	98.9	-	-	165	91.7	-	-	< 0.001	154	99.4	-	-	197	92.5	-	-	0.002
	Yes	2	1.1	-	-	15	8.3	-	-	-	1	0.6	-	-	16	7.5	-	-	-

HIV, human immunodeficiency virus.

### Regression results

The logistic regression models are presented in Table 3, with being male again being significantly associated with current cannabis and lifetime cannabis use (OR = 4.90, 95% CI 2.49–9.62 and OR = 6.27, 95% CI 3.28–11.95 respectively). Current alcohol use was associated CCU (OR = 3.06, 95% CI 1.78–5.28), and being 45 years and older was associated with a lower odds of cannabis use (OR = 0.30, 95% CI 0.09–0.96)

**TABLE 3:** Logistic regression: Association between socio-demographic and clinical variables of people admitted with schizophrenia spectrum and other psychotic disorders and cannabis use.

Variable	Model 1				Model 2				Model 3				Model 4			
	Current cannabis use				Lifetime cannabis use				Current cannabis use				Lifetime cannabis use			
**Age category**																
[< 25]	-	-	-	-	-	-	-	-	-	-		-	-	-	-	-
25–34	1.13	0.37	0.60	2.13	1.31	0.46	0.65	2.62	1.14	0.37	0.60	2.15	1.32	0.47	0.66	2.64
35–44	0.42	0.19	0.17	1.04	0.46	0.22	0.18	1.16	0.42	0.19	0.17	1.04	0.46	0.22	0.18	1.16
45+	0.63	0.36	0.20	1.94	0.30	0.18	0.09	0.96	0.62	0.36	0.20	1.91	0.29	0.17	0.09	0.93
**Gender**																
[Female]	-	-	-	-	-	-	-	-	-	-	-	-	-	-	-	-
Male	4.90	1.69	2.49	9.62	6.27	2.06	3.28	11.95	4.94	1.70	2.52	9.70	6.33	2.09	3.32	12.07
**Marital status**																
[Single or divorced or widowed]	-	-	-	-	-	-	-	-	-	-	-	-	-	-	-	-
Married	0.77	0.45	0.24	2.45	0.71	0.42	0.23	2.24	0.79	0.46	0.25	2.50	0.75	0.43	0.24	2.33
**Edu cation**																
[None or remedial school]	-	-	-	-	-	-	-	-	-	-	-	-	-	-	-	-
Mainstream- highest grade	1.65	1.51	0.27	9.95	2.39	2.33	0.35	16.15	1.66	1.53	0.27	10.07	2.44	2.38	0.36	16.52
Tertiary level	1.09	1.10	0.15	7.88	1.81	1.92	0.23	14.46	1.12	1.13	0.16	8.08	1.91	2.02	0.24	15.23
**Employment**																
[Unemployed]	-	-	-	-	-	-	-	-	-	-	-	-	-	-	-	-
Employed	1.01	0.45	0.42	2.42	0.70	0.32	0.28	1.72	1.01	0.45	0.42	2.40	0.68	0.31	0.28	1.67
**Readmission**																
[No]	-	-	-	-	-	-	-	-	-	-	-	-	-	-	-	-
Yes	0.83	0.27	0.44	1.58	1.30	0.45	0.67	2.56	0.83	0.27	0.44	1.57	1.29	0.44	0.66	2.52
Duration of illness	1.00	0.01	0.99	1.00	1.00	0.01	1.00	1.00	1.00	0.01	0.99	1.00	1.00	0.01	1.00	1.00
**HIV status**																
[Positive]	-	-	-	-	-	-	-	-	-	-	-	-	-	-	-	-
Negative	1.39	0.63	0.57	3.38	1.98	0.88	0.82	4.75	1.42	0.64	0.59	3.45	2.03	0.91	0.85	4.89
Unknown	1.34	0.66	0.52	3.50	1.33	0.65	0.52	3.45	1.37	0.67	0.53	3.57	1.37	0.66	0.53	3.54
**Current alcohol use**																
[No]	-	-	-	-	-	-	-	-	-	-	-	-	-	-	-	-
Yes	3.06	0.85	1.78	5.28	2.59	0.77	1.44	4.65	-	-	-	-	-	-	-	-
**Current cocaine and cocaine containing substance use**																
[No]	-	-	-	-	-	-	-	-	-	-	-	-	-	-	-	-
Yes	4.70	4.08	0.86	25.75	6.13	7.01	0.65	57.66	-	-	-	-	-	-	-	-
**Current alcohol or cocaine us e**									3.17	0.86	1.86	5.40	2.74	0.80	1.54	4.86
[No]	-	-	-	-	-	-	-	-	-	-	-	-	-	-	-	-
Yes	-	-	-	-	-	-	-	-	-	-	-	-	-	-	-	-

Note: The entries in the “[ ]” in the tables are the reference or comparison groups for the regression model and therefore do not have values.

HIV, human immunodeficiency virus.

## Discussion

The main findings of this study were the high prevalence of current (48.9%) and lifetime (51.1%) cannabis use among people with schizophrenia spectrum and other psychotic disorders, and that being male, of younger age and current alcohol use were associated with ongoing cannabis use.

Our study showed that CCU in psychosis is high at 48.9% but slightly lower compared with a study conducted in the Eastern Cape Province of SA, which showed a prevalence of 59.8%.^19^ This difference may have been attributed to the Eastern Cape cohort being individuals with FEP only. Temmingh and Mall, in their Western Cape cohort, reported a lower prevalence of 34.3%, which may be because of their assessing for substance use disorder rather than cannabis use.^15^ In the Western Cape Province (SA), 57% of adolescents presenting with FEP were reported to be cannabis users.^21^

Paruk et al.^20^ reported the prevalence of cannabis use at 68.8% in adolescents with FEP in KZN, while Burns et al.^36^ reported 35% prevalence of comorbid cannabis use in their adult FEP sample. This study was also conducted in KZN but included patients 16–45 years of age, and it is possible that patients may be less likely to continue cannabis use with advancing age, as was evident in our study, where those 45 years and older had lower odds of cannabis use compared with the 16–34 years age group. This could be related to peer pressure and stress or negative life events experienced by younger people with psychosis.^37^ The literature also suggests that cannabis exposure during the adolescence period, a critical time in neurodevelopment, may have lasting adverse effects that predispose to the development of psychosis as the individual grows older, even when they are no longer using cannabis^38,39,40^; hence the finding of increased use in younger people is a concern and we need to ensure that young people at risk of or presenting with psychosis receive psychoeducation and care for comorbid substance use.

Similar to our findings, other studies have also consistently reported cannabis users in populations with psychosis to be predominantly male, of young age, having a low level of education and unemployed.^5,15,16,19,24,25,26^ In a USA community hospital cohort, the findings were that being male, young, unemployed and homeless was significantly associated with cannabis use in patients with schizophrenia spectrum disorders.^41^ A study conducted in Spain in patients with FEP also found users to be predominantly male who started cannabis use earlier in life.^42^ A study conducted in Morocco also reported patients diagnosed with schizophrenia who were using cannabis were young, male, presented earlier in life and had poor adherence to medication.^43^ Local findings have also been consistent, with cannabis users being more likely to be young and male.^5,19^ The vulnerability of young males suggests the need for earlier public health programmes on substance use among this cohort who may be at risk of psychosis to improve outcomes.

Our study found that 52.8% of current cannabis users were also using alcohol, which is consistent with an international study conducted in Canada, where people with psychosis were likely to have an alcohol and cannabis use disorders at 21% and 27%, respectively. Rates of substance use were highest in the first five years of psychotic illness at 20% – 33% for alcohol and 21% – 45% for cannabis. Alcohol use was associated with higher social functioning and less negative symptoms,^10^ this being similar in a study conducted in the Eastern Cape, SA; which found that patients with FEP used cannabis followed by alcohol use.^19^ This is also supported by a study conducted in the USA, which found that patients with schizophrenia spectrum disorders and cannabis use were more likely to use other substances, such as alcohol, tobacco and cocaine.^41^ It suggests the need to screen those with psychosis for polysubstance use and reinforces the call for the much-needed dual diagnosis units that cater to substance use rehabilitation and psychosis, which are not available in KZN, SA.

The finding that almost 50% of our study population had more than three or more admissions was also concerning. While on logistic regression this study did not find any associations between cannabis use and re-hospitalisation, suggesting that the findings were compounded by other factors such as poorer access to healthcare in the province where patients were possibly not admitted to hospital and managed in the community. This finding is, however, contrary to findings in the literature where studies have consistently found that schizophrenia and continued cannabis use leads to poorer outcomes, such as a more complicated course of illness, as evidenced by frequent relapses, shorter periods of time between relapses, longer hospitalisations and more intensive psychiatric care.^5,11,19,27^ This is also consistent with both local as well as other international literature.^27,44,45^ As the literature shows that discontinuing cannabis use has improved outcomes, it becomes essential that we review the modifiable factors associated with psychosis outcome.^11^

There was a lack of association between the clinical variable HIV infection with cannabis use in people with psychosis in this study, which is consistent with another local study, but inconsistent with international research.^46,47,48^ This may be because of almost 30% of participants not having a documented HIV result, which may be attributed to poor record keeping, limited HIV testing access or the inability to provide consent, which may have affected the finding. Substance use and psychosis may increase risky sexual behaviour and hence HIV risk^49,50,51^; therefore, the association between HIV and cannabis use in those living with psychosis needs to be explored further.

### Limitations

The limitations of this study include its retrospective nature, where the data rely on the quality of record keeping, which may not be consistent or detailed well. This was a hospital-based study, which may introduce sample bias and limit generalisability to community samples. In addition, cannabis use reporting was subjective from the patient and collateral information available and not verified by urine toxicology.

However, the strength of study is that this study reflects the persistent high prevalence of cannabis use and that young males remain most vulnerable. There is a need for programmes promoting greater awareness on potential harms of cannabis use in young people particularly those who may be at risk for psychosis.

## Conclusion

The high rates of cannabis use, with almost half the people in the study with psychosis still reporting current use, is an important public health concern. Cannabis use was associated with a vulnerable group of young males with lower education and comorbid alcohol use, and with over 50% having more than three or more admissions; the data suggest that its continuous use leads to a poorer prognosis. The findings highlight the need to review treatment programmes offered at psychiatry units, with an urgent need for dual diagnosis programme that addresses substance use in people living with psychosis in a comprehensive and integrated manner.

## Recommendations

Further longitudinal studies are required to better understand association between ongoing cannabis use and psychosis outcomes, including intervention studies. Clinically we need to address cannabis use with a more concerted effort and to develop dual diagnosis units to address comorbid substance use.
